# Assessment of Blood Tumor Mutational Burden as a Potential Biomarker for Immunotherapy in Patients With Non–Small Cell Lung Cancer With Use of a Next-Generation Sequencing Cancer Gene Panel

**DOI:** 10.1001/jamaoncol.2018.7098

**Published:** 2019-02-28

**Authors:** Zhijie Wang, Jianchun Duan, Shangli Cai, Miao Han, Hua Dong, Jun Zhao, Bo Zhu, Shuhang Wang, Minglei Zhuo, Jianguo Sun, Qiming Wang, Hua Bai, Jiefei Han, Yanhua Tian, Jing Lu, Tongfu Xu, Xiaochen Zhao, Guoqiang Wang, Xinkai Cao, Fugen Li, Dalei Wang, Yuejun Chen, Yuezong Bai, Jing Zhao, Zhengyi Zhao, Yuzi Zhang, Lei Xiong, Jie He, Shugeng Gao, Jie Wang

**Affiliations:** 1 State Key Laboratory of Molecular Oncology, Department of Medical Oncology, National Cancer Center/Cancer Hospital, Chinese Academy of Medical Sciences & Peking Union Medical College, Beijing, China; 2 The Medical Department, 3D Medicines Inc, Shanghai, China; 3 The Bioinformatics Department, R&D Center of Precision Medicine, 3D Medicines Inc, Shanghai, China; 4 Department of Thoracic Medical Oncology, Peking University School of Oncology, Beijing Cancer Hospital & Institute, Beijing, China; 5 Cancer Institute, Xinqiao Hospital, Army Medical University, Chongqing, China; 6 GCP Center, National Cancer Center/Cancer Hospital, Chinese Academy of Medical Sciences & Peking Union Medical College, Beijing, China; 7 Department of Internal Medicine, Affiliated Cancer Hospital of Zhengzhou University, Henan Cancer Hospital, Zhengzhou, China; 8 The 3DMed Clinical Laboratory, 3D Medicines Inc, Shanghai, China

## Abstract

**Importance:**

Tumor mutational burden (TMB), as measured by whole-exome sequencing (WES) or a cancer gene panel (CGP), is associated with immunotherapy responses. However, whether TMB estimated by circulating tumor DNA in blood (bTMB) is associated with clinical outcomes of immunotherapy remains to be explored.

**Objectives:**

To explore the optimal gene panel size and algorithm to design a CGP for TMB estimation, evaluate the panel reliability, and further validate the feasibility of bTMB as a clinical actionable biomarker for immunotherapy.

**Design, Setting, and Participants:**

In this cohort study, a CGP named NCC-GP150 was designed and virtually validated using The Cancer Genome Atlas database. The correlation between bTMB estimated by NCC-GP150 and tissue TMB (tTMB) measured by WES was evaluated in matched blood and tissue samples from 48 patients with advanced NSCLC. An independent cohort of 50 patients with advanced NSCLC was used to identify the utility of bTMB estimated by NCC-GP150 in distinguishing patients who would benefit from anti–programmed cell death 1 (anti–PD-1) and anti–programmed cell death ligand 1 (anti–PD-L1) therapy. The study was performed from July 19, 2016, to April 20, 2018.

**Main Outcomes and Measures:**

Assessment of the Spearman correlation coefficient between bTMB estimated by NCC-GP150 and tTMB calculated by WES. Evaluation of the association of bTMB level with progression-free survival and response to anti–PD-1 and anti–PD-L1 therapy.

**Results:**

This study used 2 independent cohorts of patients with NSCLC (cohort 1: 48 patients; mean [SD] age, 60 [13] years; 15 [31.2%] female; cohort 2: 50 patients; mean [SD] age, 58 [8] years; 15 [30.0%] female). A CGP, including 150 genes, demonstrated stable correlations with WES for TMB estimation (median *r*^2^ = 0.91; interquartile range, 0.89-0.92), especially when synonymous mutations were included (median *r*^2^ = 0.92; interquartile range, 0.91-0.93), whereas TMB estimated by the NCC-GP150 panel found higher correlations with TMB estimated by WES than most of the randomly sampled 150-gene panels. Blood TMB estimated by NCC-GP150 correlated well with the matched tTMB calculated by WES (Spearman correlation = 0.62). In the anti–PD-1 and anti–PD-L1 treatment cohort, a bTMB of 6 or higher was associated with superior progression-free survival (hazard ratio, 0.39; 95% CI, 0.18-0.84; log-rank *P* = .01) and objective response rates (bTMB ≥6: 39.3%; 95% CI, 23.9%-56.5%; bTMB <6: 9.1%; 95% CI, 1.6%-25.9%; *P* = .02).

**Conclusions and Relevance:**

The findings suggest that established NCC-GP150 with an optimized gene panel size and algorithm is feasible for bTMB estimation, which may serve as a potential biomarker of clinical benefit in patients with NSCLC treated with anti–PD-1 and anti–PD-L1 agents.

## Key Points

**Question:**

Is blood tumor mutational burden estimated by a next-generation gene sequencing panel with an optimized panel size and algorithm associated with clinical outcomes in patients with non–small cell lung cancer treated with anti–programmed cell death 1 (anti–PD-1) and anti–programmed cell death ligand 1 (anti–PD-L1) agents?

**Findings:**

This study of 2 independent cohorts of patients (48 in cohort 1 and 50 in cohort 2) found that NCC-GP150 was a cost-effective panel for tumor mutational burden estimation with satisfactory performance. Blood tumor mutational burden estimated by NCC-GP150 correlated well with tissue tumor mutational burden calculated by whole-exome sequencing, and a blood tumor mutational burden of 6 or higher was positively associated with clinical benefits of anti–PD-1 and anti–PD-L1 therapy in patients with advanced non–small cell lung cancer.

**Meaning:**

The findings suggest that blood tumor mutational burden measured by NCC-GP150 is a potential biomarker to identify patients with non–small cell lung cancer who could benefit from anti–PD-1 and anti–PD-L1 therapy.

## Teaser

This study explores whether blood tumor mutational burden estimated by a next-generation sequencing gene panel is associated with clinical outcomes of patients with non–small cell lung cancer treated with anti–programmed cell death 1 and anti–programmed cell death ligand 1 agents.

## Introduction

High tumor mutational burden (TMB), which represents genomic instability, has the potential to induce neoantigen production and further immunogenicity improvement.^1^ Recent studies have confirmed that TMB measured by whole-exome sequencing (WES) or a next-generation sequencing (NGS) cancer gene panel (CGP) can serve as a candidate biomarker of clinical outcome from immune checkpoint blockades (ICBs)^2,3^ in melanoma,^4,5^ lung cancer,^6,7,8,9^ and urothelial carcinoma.^10^ However, a considerable proportion of patients with advanced cancer could not provide sufficient tumor tissue for molecular testing.^8,11^ Therefore, whether TMB can be measured using circulating tumor DNA (ctDNA) (namely, blood TMB [bTMB]) as a noninvasive approach to guide ICB therapies has attracted widespread attention from practitioners. A previous study^12^ found that blood-derived variants of unknown significance were correlated with ICB responses. More recently, Gandara et al^13^ reported that bTMB is associated with progression-free survival (PFS) benefit from atezolizumab over docetaxel in non–small cell lung cancers (NSCLCs). Further evaluation of bTMB will be performed in patients undergoing first-line treatment in a prospective, phase 3 randomized clinical trial (A Study of Atezolizumab as First-line Monotherapy for Advanced or Metastatic Non–Small Cell Lung Cancer [B-F1RST]).^14^ However, the reliability of ctDNA detection is still under debate^15,16^; thus, more supported evidence of bTMB is needed to promote its clinical value on guiding ICB delivery. In this study, we aimed to explore the optimal gene panel size and algorithm to design a CGP for TMB estimation, evaluate the panel reliability, and further validate the feasibility of bTMB as a clinically actionable biomarker for immunotherapy.

## Methods

### Study Design

This study contained 4 sections (eFigure 1 in the Supplement), including panel design (named NCC-GP150), virtual validation (eMethods 1 in the Supplement), technical validation, and clinical validation (eMethods 2 in the Supplement). The WES data from The Cancer Genome Atlas (TCGA) were used for panel design and virtual validation. Tumor mutational burden estimated by NCC-GP150 was compared with those by established gene panels, including Memorial Sloan Kettering Cancer Center’s Integrated Mutation Profiling of Actionable Cancer Targets (MSK-IMPACT), FoundationOne CDx (F1CDx), Guardant360, PlasmaSELECT 64, and FoundationACT (Assay for Circulating Tumor DNA). A public NSCLC cohort from Rizvi et al^6^ was used to evaluate the performance of NCC-GP150–based TMB to stratify ICB survival outcomes. Patients with NSCLC with sufficient tumor tissue samples and matched plasma samples were enrolled for technical validation to investigate the correlation between bTMB from the NCC-GP150 panel and tTMB from WES (eFigure 2A in the Supplement). Last, an independent cohort of patients with advanced NSCLC who were undergoing on-study anti–programmed cell death 1 (anti–PD-1) and anti–programmed cell death ligand 1 (anti–PD-L1) therapy was analyzed to validate the utility of bTMB by NCC-GP150 in identifying patients who could benefit from anti–PD-1 and anti–PD-L1 therapy (eFigure 2B and eMethods 3 in the Supplement). The study was performed from July 19, 2016, to April 20, 2018. For more details about the methods for DNA extraction, library preparation, target capture and DNA sequencing, WES analysis pipeline, and bTMB detection pipeline, see eMethods 4 to 7 in the Supplement. This study was approved by the ethics committees of the National Cancer Center, and all patients provided written informed consent. All data were deidentified.

### Statistical Analysis

Correlations of TMB between WES and gene panels with different gene counts and algorithms were examined by the Pearson correlation coefficient (*r*^2^). The correlation between tissue-based TMB by WES and ctDNA-based bTMB by NCC-GP150 was determined by the Spearman rank correlation coefficient. Categorical variables were expressed as percentages, means (SDs) were provided for normally distributed data, and medians (interquartile ranges) were provided for data that are not normally distributed. Differences between the 2 groups were examined by the 2-tailed, unpaired *t* test for normally distributed variables or by the Mann-Whitney test for nonnormally distributed variable. The χ^2^ test or Fisher exact test was used to test the difference of categorical variables between the 2 groups. For PFS analysis, Kaplan-Meier curves were compared by using a log-rank test, and the hazard ratio (HR) was determined through a Cox proportional hazards regression model. The proportionality assumption was verified. Logistic regression was used to test the correlations between different variables and the objective response rate (ORR), with the results presented as odds ratios (ORs) and 95% CIs. Baseline variables that achieved a level of significance of *P* < . 05 in the univariable analysis were entered into multivariable models. All reported *P* values were 2-tailed, and *P* < .05 was considered to be statistically significant. Statistical analyses were performed with GraphPad Prism software, version 5.0 (GraphPad Software Inc) and R software, version 3.5.0 (R Foundation for Statistical Computing).

## Results

This study used 2 independent cohorts of patients with NSCLC (cohort 1: 48 patients; mean [SD] age, 60 [13] years; 15 [31.2%] female; cohort 2: 50 patients; mean [SD] age, 58 [8] years; 15 [30.0%] female) to examine the correlation between bTMB estimated by NCC-GP150 and tTMB measured by WES and to identify the utility of bTMB estimated by NCC-GP150 in distinguishing patients who would benefit from anti–PD-1 and anti–PD-L1 therapy.

### Correlation of the NCC-GP150 Panel With TCGA-Based WES Data for TMB Estimation

**Figure 1.**
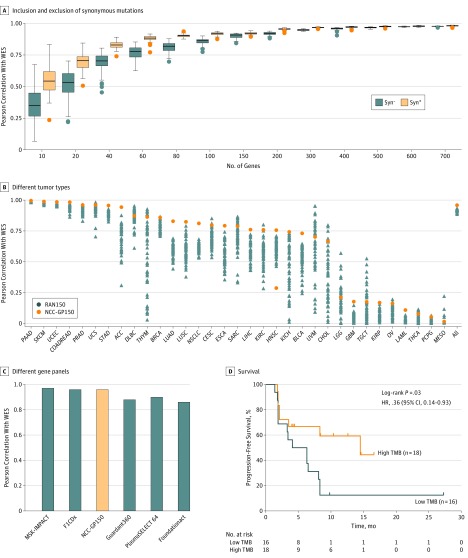
Panel Design and Virtual Validation of the Association Between Blood and Tissue Tumor Mutational Burden (TMB) A, Distribution of Pearson correlations between whole-exome sequencing (WES) and different numbers of genes randomly chosen (50 times) from gene panels. Syn + and Syn − indicate that synonymous mutations were included or excluded, respectively. B, Comparison of the performance of the NCC-GP150 panel and 150 randomly extracted genes (RAN150) among different tumor types. C, Pearson correlation of TMB between different public cancer gene panels and WES. D, Progression-free survival (PFS) by TMB status based on NCC-GP150 genes in the cohort studied by Rizvi et al.^6^ F1CDx indicates FoundationOne CDx; MSK-IMPACT, Memorial Sloan Kettering Cancer Center’s Integrated Mutation Profiling of Actionable Cancer Targets.

With the WES data of 9205 samples from TCGA, randomized genes were extracted to generate panels for TMB estimation and compared with WES-based TMB. With the increase in randomized gene number included in TMB calculation, a gradually increasing correlation between the panel- and WES-based TMB was observed along with a decreasing SD, reaching a plateau when 150 genes were included (Figure 1A). An NGS CGP, named NCC-GP150, was designed that covered whole exon regions of 150 selected cancer-related genes (eTable 1 in the Supplement). NCC-GP150 exhibited a better performance than most of the randomly sampled panels in most cancer types based on TCGA data (Figure 1B).

Subsequently, NCC-GP150 was compared with 5 established NGS gene panels, including MSK-IMPACT (468 cancer-related genes), F1CDx (324 cancer-related genes), Guardant360 (73 cancer-related genes), PlasmaSELECT 64 (64 cancer-related genes), and FoundationACT (62 cancer-related genes). The analysis of overlapping genes among these panels is shown in eFigure 3 in the Supplement. Among these gene panels, MSK-IMPACT exhibited a leading correlation with WES-based TMB (*r*^2^ = 0.97), followed by F1CDx (*r^2^* = 0.96) and NCC-GP150 (*r^2^* = 0.96) (Figure 1C). The performance of NCC-GP150 for TMB estimation in various cancers by virtual validation is given in eTable 2 in the Supplement.

Considering the different prevalence of oncogenic driver mutations in NSCLC between Asian and white populations,^17^ we conducted another virtual validation of NCC-GP150 with established panels between TCGA NSCLC populations with and without *EGFR* (OMIM 131550) and/or *KRAS* (OMIM 190070) driver mutation. The NCC-GP150 demonstrated a consistently satisfactory performance for bTMB estimation vs TMB from TCGA WES data (eFigure 4 in the Supplement).

Next, we used a published clinical data set^6^ that included 34 patients with NSCLC treated with PD-1 to further test the practicability of NCC-GP150. The PFS was significantly longer in patients with high TMB (TMB greater than the median: PFS, 14.5 months; 95% CI, 8.3 months to not reached [NR]) than in patients with low TMB (TMB less than the median: PFS, 5.2 months; 95% CI, 2.1-8.3 months), with an HR of 0.36 (95% CI, 0.14-0.93, log-rank *P* = .03) (Figure 1D).

### Correlation of bTMB Estimated by NCC-GP150 With tTMB Calculated by WES

To investigate the reliability of bTMB from ctDNA-derived sequencing by the NCC-GP150 panel, 48 patients with advanced NSCLC with qualified tumor tissue samples and matched plasma samples provided for synchronous WES and NCC-GP150 sequencing, respectively, were enrolled for technical validation (cohort 1) (eFigure 2A and eTable 3 in the Supplement). The Spearman correlation coefficient between NCC-GP150–based bTMB and WES-based TMB reached 0.62 (eFigure 5A in the Supplement). With the TMB median (75 for WES) as the cut point, we found that a bTMB of 6 or higher had an optimal Youden index of 0.59, with a sensitivity of 0.88 and a specificity of 0.71 (eFigure 5B and eTable 4 in the Supplement).

### bTMB Estimated by the NCC-GP150 Panel and Clinical Outcomes of NSCLC Treated With ICBs

To unravel whether bTMB could identify patients benefiting from ICB therapy, another independent cohort of 50 patients with advanced NSCLC treated with anti–PD-1 and anti–PD-L1 agents (cohort 2) (eFigure 2B and eTable 5 in the Supplement) was used for analysis. Methods for the assessment of clinical outcomes are provided in eMethods 3 in the Supplement.

**Figure 2.**
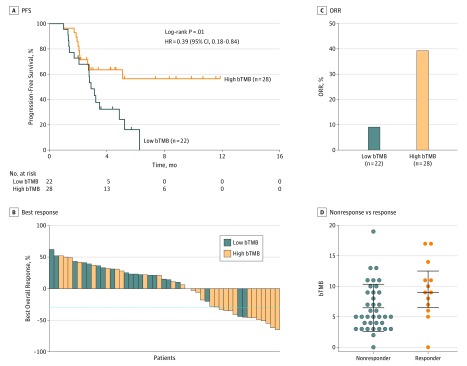
Clinical Validation of the Association Between NCC-GP150–Derived Blood Tumor Mutational Burden (bTMB) and Clinical Benefit in Patients With Non–Small Cell Lung Cancer A, Progression-free survival (PFS) by bTMB status. B, Waterfall plot of observed best response from anti–programmed cell death 1 (anti–PD-1) and anti–programmed cell death ligand 1 (anti–PD-L1) checkpoint inhibitors. C, Comparison of objective response rates (ORRs) between the high and low bTMB groups (*P* = .02). D, Comparison of bTMB level between nonresponse and response groups (*P* = .02). HR indicates hazard ratio.

The bTMB and PD-L1 expression were not correlated (eTable 5 in the Supplement), which is consistent with previous results.^8,13,18,19^ When the bTMB cut point was set to 6, both HRs and *P* values reached a minimum (eFigure 6 in the Supplement). Compared with patients with low bTMB (bTMB<6, n = 22), patients with high bTMB (bTMB≥6, n = 28) demonstrated superior PFS (high bTMB: PFS, NR; 95% CI, 2.8 months to NR; low bTMB: PFS, 2.9 months; 95% CI, 2.7 months to NR; HR, 0.39; 95% CI, 0.18-0.84; log-rank *P* = .01) (Figure 2A) and were more likely to undergo tumor shrinkage (Figure 2B). In addition, high bTMB (39.3%; 95% CI, 23.9%-56.5%) was associated with a higher ORR than was low bTMB (9.1%; 95% CI, 1.6%-25.9%; *P* = .02) (Figure 2C). Similarly, responders had significantly higher bTMB levels than did nonresponders (Mann-Whitney *P* = .02) (Figure 2D).

In the univariable Cox proportional hazards regression model, the Eastern Cooperative Oncology Group (ECOG) and treatment lines were also associated with PFS (ECOG: HR, 2.67; 95% CI, 1.21-5.88; *P* = .02; treatment lines: HR, 4.50; 95% CI, 2.05-9.89; *P* < .001), and the association between PD-L1 of 1% or higher and benefits for immunotherapy tended to be significant (HR, 0.49; 95% CI, 0.21-1.15; *P* = .10) (Table). In the multivariate Cox proportional hazards regression model that included bTMB, ECOG, and treatment lines, the association between bTMB and PFS remained significant (HR, 0.44; 95% CI, 0.20-0.99; *P* = .05). In the multivariate logistic regression analysis that included ECOG and treatment lines, bTMB status was also positively associated with an ORR (odds ratio, 11.69; 95% CI, 2.16-111.6; *P* = .01) (Table).

**Table.** Univariable and Multivariable Analysis of Progression-Free Survival and Objective Response Rates^a^

Parameter	Progression-Free Survival				Objective Response Rate			
	Univariable Analysis		Multivariable Analysis		Univariable Analysis		Multivariable Analysis	
	HR (95% CI)	*P* Value	HR (95% CI)	*P* Value	OR (95% CI)	*P* Value	OR (95% CI)	*P* Value
Age ≥65 vs <65 y	0.62 (0.21-1.79)	.37	NA	NA	2.84 (0.60-13.12)	.17	NA	NA
Male vs female	0.62 (0.28-1.34)	.22	NA	NA	2.98 (0.67-21.17)	.20	NA	NA
ECOG performance status ≥2 vs 1 or 0	2.67 (1.21-5.88)	.02	2.31 (1.08-4.95)	.03	0.46 (0.12-1.57)	.23	0.35 (0.04-1.89)	.25
≥3 vs <3 Metastatic sites	0.83 (0.39-1.75)	.62	NA	NA	1.23 (0.34-4.51)	.75	NA	NA
LDH≥250 vs <250 U/L	1.19 (0.55-2.55)	.66	NA	NA	1.30 (0.33-4.80)	.69	NA	NA
PD-L1 status ≥1% vs <1%	0.49 (0.21-1.15)	.10	NA	NA	2.47 (0.49-18.6)	.31	NA	NA
Current or former vs never smoker	0.86 (0.41-1.80)	.69	NA	NA	1.69 (0.47-6.51)	.43	NA	NA
bTMB≥6 vs <6	0.39 (0.18-0.84)	.02	0.44 (0.20-0.99)	.05	6.47 (1.48-45.72)	.03	11.69 (2.16-111.6)	.01
≥3 vs 1 or 2 Lines of PD-1/PD-L1 blocked therapy	4.50 (2.05-9.89)	<.001	3.34 (1.50-7.43)	.003	0.11 (0.01-0.64)	.04	0.11 (0.006-0.79)	.06

Abbreviations: bTMB, blood tumor mutational burden; ECOG, Eastern Cooperative Oncology Group; HR, hazard ratio; LDH, lactate dehydrogenase; NA, not applicable; OR, odds ratio; PD-1, programmed cell death 1; PD-L1, programmed cell death ligand 1.

SI conversion factor: To convert lactate dehydrogenase to microkatals per liter, multiply by 0.0167.

^a^ Baseline variables that achieved a level of significance of *P* < .05 in the univariable analysis were entered into multivariable models.

We further explored the association between bTMB and PFS in first-line or second-line NSCLC as a subgroup analysis. The HRs and *P* values still reached a minimum, with a high bTMB cut point of 6 (HR, 0.08; 95% CI, 0.02-0.36; *P* = .001) (eFigure 7 in the Supplement). Patients with a bTMB of 6 or higher in this subgroup yielded significantly prolonged PFS (NR) compared with patients with a bTMB of less than 6 (2.9 months; 95% CI, 2.7 months to NR; HR, 0.08; 95% CI, 0.02-0.36; log-rank *P* < .001) (eFigure 8A in the Supplement). In addition, patients with a bTMB of 6 or higher had an increased ORR (61.1%; 95% CI, 39.2%-80.1%) compared with patients with a bTMB less than 6 (6.7%, 95% CI, 0.3%-27.9%; *P* = .003) (eFigure 8B in the Supplement). Responders had significantly higher bTMB levels (median, 10; interquartile range, 7-13) than did nonresponders (median, 5; interquartile range, 3-9; Mann-Whitney *P* = .008) (eFigure 8C in the Supplement). Collectively, bTMB measured by the NCC-GP150 panel was confirmed to be a potential clinical actionable biomarker for ICB therapy in patients with NSCLC.

## Discussion

Our findings suggest that NCC-GP150, a panel through rational design on TCGA, could be used for bTMB estimation as a surrogate for WES-based TMB. We also validated bTMB as a potential biomarker to identify patients with NSCLC who could obtain significant improvements from immunotherapy.

Blood TMB profiled with ctDNA sequencing is a promising strategy for TMB and ICB response estimation. Most recently, Gandara et al^13^ published the first study, to our knowledge, to identify bTMB of 16 or higher as an indicator of PFS benefit in patients with NSCLC treated with atezolizumab vs docetaxel. However, several key questions remained to be answered, including the suitable panel size and the kind of variants that should be included for TMB calling. In addition, in the reported study, analyses were made between bTMB and tTMB that were derived from the same gene panel but not with WES-based TMB as the criterion standard.

We present the first study, to our knowledge, to systematically explore the optimal gene panel size and algorithm of a CGP design for TMB (especially bTMB) estimation. With WES data from TCGA, we found that a minimum gene panel size of 150 was sufficient for TMB estimation. The NCC-GP150 panel was then established through rational design with 150 selected genes and performed at the forefront for TMB estimation among most random-sampling models. We also found that the incorporation of synonymous mutations into panel-derived TMB calculation enhanced its correlation with WES, which agrees with the previous finding.^20^ Furthermore, in our independent cohort 1, we validated the satisfactory correlation of NCC-GP150–based bTMB with WES-based tTMB. Taken together, NCC-GP150 with a smaller panel size and satisfactory performance may be more accessible for clinic use with superior cost-effectiveness.

Our study also found that bTMB may be a potential biomarker to differentiate patients benefiting from anti–PD-1 and anti–PD-L1 therapy. The utility of the NCC-GP150 panel seemed to be more significant when ICBs were used as a first- or second-line rather than a later-line treatment. In addition, ECOG performance status and treatment lines were found to be negatively correlated with PFS, suggesting that patients with previous treatment might receive limited benefit from ICB therapy, which agrees with previous reports.^21,22^ Therefore, early use of therapy with ICBs should be considered in clinical practice.

### Limitations

There are several limitations to our study. First, the clinical validation was retrospective, and the limited sample sizes might yield statistical bias. Second, the clinical cohort was obtained from different trials, including anti–PD-1 and anti–PD-L1 treatment, which might potentially influence the ultimate survival outcomes. Third, we used TCGA data for virtual validation but a Chinese cohort for technical and clinical validation. The different population-based prevalence in oncogenic driver mutations may have a potential confounding effect on the validity of analyses,^17^ although NCC-GP150 still demonstrated consistently satisfactory performance for TCGA based-TMB estimation and Chinese cohort–based bTMB estimation after excluding patients with *EGFR*/*KRAS* driver mutations.

## Conclusions

We developed and evaluated the clinical feasibility of the NCC-GP150 panel for TMB estimation through virtual, technical, and clinical validation. The findings suggest that a ctDNA-based bTMB measured by the NCC-GP150 panel could be used as a potential biomarker for anti–PD-1 and anti–PD-L1 treatment in patients with NSCLC.

## Notes

Supplement. **eMethods 1.** Gene Panel Design and Virtual Validation **eMethods 2.** Technical Validation and Clinical Validation **eMethods 3.** Assessment of Clinical Outcomes **eMethods 4.** DNA Extraction **eMethods 5.** Library Preparation, Target Capture and DNA Sequencing **eMethods 6.** WES Analysis Pipeline **eMethods 7.** bTMB Detection Pipeline **eFigure 1.** Flow Diagram of the Study **eFigure 2.** Flow Diagram for the Technical and Clinical Validation Cohorts **eFigure 3.** Venn Diagram and Flow Diagram Showing the Numbers and Percentages of Overlapping Gene Numbers Among NCC-GP150, F1CDx, MSK-IMPACT, and Guardant360 **eFigure 4.** Comparison of Panel Performance Between TCGA NSCLC Populations With and Without EGFR or KRAS Driver Mutation **eFigure 5.** Technical Validation of the Association Between bTMB Estimated by NCC-GP150 and WES **eFigure 6.** Comparison of Progression-Free Survival Between Patients in the bTMB-H and bTMB-L Groups With Different Cut-Points for bTMB **eFigure 7.** Comparison of Progression-Free Survival Between bTMB-H and bTMB-L With Different Cut-Points for bTMB in Patients With Anti-PD1/PD-L1 Therapy as First- or Second-Line Treatment **eFigure 8.** Association Between bTMB and Clinical Outcomes in Patients With Anti-PD1/PD-L1 Therapy as First- or Second-Line Treatment **eTable 1.** Detailed list of Genes in the NCC-GP150 Panel **eTable 2.** Summary of Pearson Correlation Between Different Public Panels and WES for Different Tumor Types **eTable 3.** Detailed Clinicopathologic Features of 48 NSCLC Patients for bTMB Technical Validation **eTable 4.** Summary of Sensitivity, Specificity and Youden's Index at Different Cutoffs for bTMB-H in the Matched Tissue/Blood TMB Comparison **eTable 5.** Detailed Clinicopathologic Features of 50 NSCLC Patients for bTMB Clinical Validation by bTMB Status **eReferences**

## References

[1] Schumacher TN, Schreiber RD Neoantigens in cancer immunotherapy. Science. 2015;348(6230):69-74. doi:10.1126/science.aaa4971 25838375

[2] Yarchoan M, Johnson BA III, Lutz ER, Laheru DA, Jaffee EM Targeting neoantigens to augment antitumour immunity. Nat Rev Cancer. 2017;17(4):209-222. doi:10.1038/nrc.2016.154 28233802 PMC5575801

[3] Yarchoan M, Hopkins A, Jaffee EM Tumor mutational burden and response rate to PD-1 inhibition. N Engl J Med. 2017;377(25):2500-2501. doi:10.1056/NEJMc1713444 29262275 PMC6549688

[4] Snyder A, Makarov V, Merghoub T, Genetic basis for clinical response to CTLA-4 blockade in melanoma. N Engl J Med. 2014;371(23):2189-2199. doi:10.1056/NEJMoa1406498 25409260 PMC4315319

[5] Van Allen EM, Miao D, Schilling B, Genomic correlates of response to CTLA-4 blockade in metastatic melanoma. Science. 2015;350(6257):207-211. doi:10.1126/science.aad0095 26359337 PMC5054517

[6] Rizvi NA, Hellmann MD, Snyder A, Cancer immunology: mutational landscape determines sensitivity to PD-1 blockade in non–small cell lung cancer. Science. 2015;348(6230):124-128. doi:10.1126/science.aaa1348 25765070 PMC4993154

[7] Carbone DP, Reck M, Paz-Ares L, ; CheckMate 026 Investigators First-line nivolumab in stage IV or recurrent non–small-cell lung cancer. N Engl J Med. 2017;376(25):2415-2426. doi:10.1056/NEJMoa1613493 28636851 PMC6487310

[8] Hellmann MD, Ciuleanu TE, Pluzanski A, Nivolumab plus ipilimumab in lung cancer with a high tumor mutational burden. N Engl J Med. 2018;378(22):2093-2104. doi:10.1056/NEJMoa1801946 29658845 PMC7193684

[9] Hellmann MD, Callahan MK, Awad MM, Tumor mutational burden and efficacy of nivolumab monotherapy and in combination with ipilimumab in small-cell lung cancer. Cancer Cell. 2018;33(5):853-861, e854. doi:10.1016/j.ccell.2018.04.001 29731394 PMC6750707

[10] Balar AV, Galsky MD, Rosenberg JE, ; IMvigor210 Study Group Atezolizumab as first-line treatment in cisplatin-ineligible patients with locally advanced and metastatic urothelial carcinoma: a single-arm, multicentre, phase 2 trial. Lancet. 2017;389(10064):67-76. doi:10.1016/S0140-6736(16)32455-2 27939400 PMC5568632

[11] Kim ES, Hirsh V, Mok T, Gefitinib versus docetaxel in previously treated non-small-cell lung cancer (INTEREST): a randomised phase III trial. Lancet. 2008;372(9652):1809-1818. doi:10.1016/S0140-6736(08)61758-4 19027483

[12] Khagi Y, Goodman AM, Daniels GA, hypermutated circulating tumor DNA: correlation with response to checkpoint inhibitor-based immunotherapy. Clin Cancer Res. 2017;23(19):5729-5736. doi:10.1158/1078-0432.CCR-17-1439 28972084 PMC5678984

[13] Gandara DR, Paul SM, Kowanetz M, Blood-based tumor mutational burden as a predictor of clinical benefit in non-small-cell lung cancer patients treated with atezolizumab. Nat Med. 2018;24(9):1441-1448. doi:10.1038/s41591-018-0134-3 30082870

[14] Kim ES, Velcheti V, Mekhail T, Primary efficacy results from B-F1RST, a prospective phase II trial evaluating blood-based tumour mutational burden (bTMB) as a predictive biomarker for atezolizumab (atezo) in 1L non-small cell lung cancer (NSCLC). Ann Oncol. 2018;29(suppl 8):mdy424.067. doi:10.1093/annonc/mdy424.067

[15] Torga G, Pienta KJ Patient-paired sample congruence between 2 commercial liquid biopsy tests. JAMA Oncol. 2018;4(6):868-870. doi:10.1001/jamaoncol.2017.4027 29242909 PMC6145681

[16] Kuderer NM, Burton KA, Blau S, Comparison of 2 commercially available next-generation sequencing platforms in oncology. JAMA Oncol. 2017;3(7):996-998. doi:10.1001/jamaoncol.2016.4983 27978570 PMC5824236

[17] Shi Y, Au JS, Thongprasert S, A prospective, molecular epidemiology study of EGFR mutations in Asian patients with advanced non-small-cell lung cancer of adenocarcinoma histology (PIONEER). J Thorac Oncol. 2014;9(2):154-162. doi:10.1097/JTO.0000000000000033 24419411 PMC4132036

[18] Rizvi H, Sanchez-Vega F, La K, Molecular determinants of response to anti-programmed cell death (PD)-1 and anti-programmed death-ligand 1 (PD-L1) blockade in patients with non-small-cell lung cancer profiled with targeted next-generation sequencing. J Clin Oncol. 2018;36(7):633-641. doi:10.1200/JCO.2017.75.3384 29337640 PMC6075848

[19] Hellmann MD, Nathanson T, Rizvi H, Genomic features of response to combination immunotherapy in patients with advanced non–small-cell lung cancer. Cancer Cell. 2018;33(5):843-852.e4. doi:10.1016/j.ccell.2018.03.018 29657128 PMC5953836

[20] Chalmers ZR, Connelly CF, Fabrizio D, Analysis of 100,000 human cancer genomes reveals the landscape of tumor mutational burden. Genome Med. 2017;9(1):34. doi:10.1186/s13073-017-0424-2 28420421 PMC5395719

[21] Fujimoto D, Yoshioka H, Kataoka Y, Efficacy and safety of nivolumab in previously treated patients with non–small cell lung cancer: a multicenter retrospective cohort study. Lung Cancer. 2018;119:14-20. doi:10.1016/j.lungcan.2018.02.017 29656747

[22] Mezquita L, Auclin E, Ferrara R, Association of the lung immune prognostic index with immune checkpoint inhibitor outcomes in patients with advanced non–small cell lung cancer. JAMA Oncol. 2018;4(3):351-357. doi:10.1001/jamaoncol.2017.4771 29327044 PMC5885829

