# Supplementary material for: Assessment of Blood Tumor Mutational Burden as a Potential Biomarker for Immunotherapy in Patients With Non–Small Cell Lung Cancer With Use of a Next-Generation Sequencing Cancer Gene Panel
Source: JAMA Oncol. 2019 Feb 28;5(5):696–702. doi: 10.1001/jamaoncol.2018.7098 (PMC6512308; doi:10.1001/jamaoncol.2018.7098)
Supplement: Supplement. — eMethods 1. Gene Panel Design and Virtual Validation eMethods 2. Technical Validation and Clinical Validation eMethods 3. Assessment of Clinical Outcomes eMethods 4. DNA Extraction eMethods 5. Library Preparation, Target Capture and DNA Sequencing eMethods 6. WES Analysis Pipeline eMethods 7. bTMB Detection Pipeline eFigure 1. Flow Diagram of the Study eFigure 2. Flow Diagram for the Technical and Clinical Validation Cohorts eFigure 3. Venn Diagram and Flow Diagram Showing the Numbers and Percentages of Overlapping Gene Numbers Among NCC-GP150, F1CDx, MSK-IMPACT, and Guardant360 eFigure 4. Comparison of Panel Performance Between TCGA NSCLC Populations With and Without EGFR or KRAS Driver Mutation eFigure 5. Technical Validation of the Association Between bTMB Estimated by NCC-GP150 and WES eFigure 6. Comparison of Progression-Free Survival Between Patients in the bTMB-H and bTMB-L Groups With Different Cut-Points for bTMB eFigure 7. Comparison of Progression-Free Survival Between bTMB-H and bTMB-L With Different Cut-Points for bTMB in Patients With Anti-PD1/PD-L1 Therapy as First- or Second-Line Treatment eFigure 8. Association Between bTMB and Clinical Outcomes in Patients With Anti-PD1/PD-L1 Therapy as First- or Second-Line Treatment eTable 1. Detailed list of Genes in the NCC-GP150 Panel eTable 2. Summary of Pearson Correlation Between Different Public Panels and WES for Different Tumor Types eTable 3. Detailed Clinicopathologic Features of 48 NSCLC Patients for bTMB Technical Validation eTable 4. Summary of Sensitivity, Specificity and Youden's Index at Different Cutoffs for bTMB-H in the Matched Tissue/Blood TMB Comparison eTable 5. Detailed Clinicopathologic Features of 50 NSCLC Patients for bTMB Clinical Validation by bTMB Status eReferences [file jamaoncol-5-696-s001.pdf]

Supplementary Online Content

Wang Z, Duan J, Cai S, et al. Assessment of blood tumor mutational burden as a potential biomarker for immunotherapy in patients with non–small cell lung cancer with use of a next-generation sequencing cancer gene panel. *JAMA Oncol.* Published online February 28, 2018. doi:10.1001/jamaoncol.2018.7098

eMethods 1. Gene Panel Design and Virtual Validation
eMethods 2. Technical Validation and Clinical Validation
eMethods 3. Assessment of Clinical Outcomes
eMethods 4. DNA Extraction
eMethods 5. Library Preparation, Target Capture and DNA Sequencing
eMethods 6. WES Analysis Pipeline
eMethods 7. bTMB Detection Pipeline
eFigure 1. Flow Diagram of the Study
eFigure 2. Flow Diagram for the Technical and Clinical Validation Cohorts
eFigure 3. Venn Diagram and Flow Diagram Showing the Numbers and Percentages of Overlapping Gene Numbers Among NCC-GP150, F1CDx, MSK-IMPACT, and Guardant360
eFigure 4. Comparison of Panel Performance Between TCGA NSCLC Populations With and Without EGFR or KRAS Driver Mutation
eFigure 5. Technical Validation of the Association Between bTMB Estimated by NCC-GP150 and WES
eFigure 6. Comparison of Progression-Free Survival Between Patients in the bTMB-H and bTMB-L Groups With Different Cut-Points for bTMB
eFigure 7. Comparison of Progression-Free Survival Between bTMB-H and bTMB-L With Different Cut-Points for bTMB in Patients With Anti-PD1/PD-L1 Therapy as First- or Second-Line Treatment
eFigure 8. Association Between bTMB and Clinical Outcomes in Patients With Anti-PD1/PD-L1 Therapy as First- or Second-Line Treatment
eTable 1. Detailed List of Genes in the NCC-GP150 Panel
eTable 2. Summary of Pearson Correlation Between Different Public Panels and WES for Different Tumor Types
eTable 3. Detailed Clinicopathologic Features of 48 NSCLC Patients for bTMB Technical Validation
eTable 4. Summary of Sensitivity, Specificity and Youden's Index at Different Cutoffs for bTMB-H in the Matched Tissue/Blood TMB Comparison
eTable 5. Detailed Clinicopathologic Features of 50 NSCLC Patients for bTMB Clinical Validation by bTMB Status
eReferences

This supplementary material has been provided by the authors to give readers additional information about their work.

© 2018 Wang Z et al. *JAMA Oncol.*

eMethods 1. Gene Panel Design and Virtual Validation

WES data of 9205 total cases across 33 cancer types from TCGA (http://www.cbioportal.org/, version 1.11.3) was applied to explore the minimal number of genes needed and whether synonymous mutations should be considered for TMB calculation. We randomly extracted genes with a genomic scope to constitute randomized gene panels ranging from 10 to 700 genes (10, 20, 40, 60, 80, 100, 150, 200, 300, 400, 500, 600 and 700). The genes included in each size panel were extracted randomly 50 times. The correlations of TMB estimated by randomized gene panels with WES were evaluated. The estimated TMB was calculated by the sum of missense, stop loss, in-frame and frameshift mutations in protein coding regions, with or without silent mutations.

Based on the virtual deduction of panel size and improved TMB calculation formula, an NGS target CGP was designed and named as NCC-GP150 that covered whole exon regions of 150 genes. Gene selections were made considering several aspects for a combination of bTMB assessment and driver gene mutation analysis to ensure maximum yield of clinically relevant information with limited sample/technical resources and economic constrains: A. Input of validated clinical actionable mutations from NCCN guidelines and published literature or database (https://cancer.sanger.ac.uk/cosmic/); B. Selection of relevant/important genes in the upstream or downstream pathways of oncogenic mutations; C. Incorporation of additional positive and negative predictors for immunotherapy; and more importantly, D. One special consideration in our panel design: Incorporation of cancer genes that were reported to be associated to tumor mutation burden, and those with high mutation recurrence across TCGA database as identified from specific cancer types with high incidences or morality rates that will contribute to the accuracy of bTMB estimation. For example, some DNA damage repair (DDR) genes were included considering these DDR mutations are prone to increased somatic mutations and associated with improved survival of immunotherapy.[1] Taken together, we have integrated genes with above features into a complete panel and fine-tuned its cost-effectiveness.

To better explore and confirm the feasibility of NCC-GP150 for TMB estimation, we analyzed the correlation between the panel and TCGA-based WES data and compared it with other virtual random-sampling models and established NGS gene panels. We also applied NCC-GP150-based TMB to the public Rizvi cohort with 34 NSCLC patients (from cBioPortal http://www.cbioportal.org/study.do?cancer_study_id=luad_mskcc_2015) to stratify TMB-high (TMB-H) and TMB-low (TMB-L) subgroups according to median value, and correlated TMB to survival outcomes.

eMethods 2. Technical Validation and Clinical Validation

To investigate the correlation of ctDNA-based bTMB estimated by NCC-GP150 and tumor-tissue-based TMB calculated by WES, we collected tumor tissue samples and paired peripheral blood samples from 66 treatment-naïve NSCLC patients diagnosed in Cancer Hospital Chinese Academy of Medical Sciences (CAMS) & Peking Union Medical College between August 1st, 2016, and January 1st, 2018. Eighteen cases were ultimately excluded due to stage I-IIIA, long collection interval between blood and tissue samples (>14 days), blood extracted after surgery, insufficient cancer cell fraction in tissue (<20%), low DNA quantitation (<50 ng), and low sequencing depth. For the remaining 48 patients, ctDNA and formalin-fixed paraffin-embedded tissue samples were used for NCC-GP150 NGS and WES sequencing, respectively. The mean depths of WES for tumor tissue and NCC-GP150 NGS for ctDNA were 239× and 3417×, respectively.

© 2018 Wang Z et al. *JAMA Oncol.*

Between July 19th, 2016 and February 27th, 2018, a total of 70 patients with stage IIIB-IV NSCLC who were undergoing on-study anti-PD-1/PD-L1 monotherapy in Cancer Hospital CAMS, Peking University Cancer Hospital and Xinqiao Hospital Army Medical University, were screened. We excluded 4 patients without efficacy evaluation due to loss to follow-up, 6 without blood samples provided within 4 weeks prior to ICB delivery, 4 with blood samples less than 8 ml, and 6 treated with combination therapy. Fifty patients were ultimately included for the analysis. All patients were still on-study or have completed the study by the cut-off date of statistical analysis on April 20, 2018. The clinical variables were extracted for each patient, including age, sex, histologic type, number of metastatic sites, ECOG performance status, smoking status, lactate dehydrogenase (LDH), PD-L1 expression, immune checkpoint blockade therapy, treatment lines, hospital sites, and survival status. The median follow-up time was 5.2 months (IQR, 4.0-9.1 months). The technicians for the generation of TMB and bTMB were blinded to the patients' clinical data. The mean depth of the targeted sequencing was 3398×.

eMethods 3. Assessment of Clinical Outcomes

Radiographic imaging was acquired by indicated approaches such as CT or MRI for tumor response assessment, which was evaluated by both the investigator and an independent radiologist. Baseline tumor assessments were performed within 1-28 days prior to the initiation of the PD-1/PD-L1 treatment, with subsequent assessments performed every 6 to 8 weeks until objective disease progression. The objective response rate (ORR) was defined as the percentage of patients with confirmed complete response (CR) or partial response (PR) by RECIST version 1.1.[2] PFS was defined as the time from the start of anti-PD-1/PD-L1 treatment until disease progression (assessed by an investigator using RECIST version 1.1) or death from any cause.

eMethods 4. DNA Extraction

The blood was centrifuged in Streck tubes at 1600 g for 20 minutes at room temperature to separate the plasma. Then, the plasma layer was carefully transferred to a new 1.5 ml Eppendorf tube, followed by room-temperature centrifugation at 16000 g for 10 minutes to remove residual cells and debris. The buffy coat was then transferred to a new tube for genomic DNA (gDNA) extraction. Tumor tissues were determined the percentage of tumor cells by H&E staining. We included in this study only samples with a tumor cell percentage >20. Afterwards, gDNA from tumor FFPE tissues and white blood cells was extracted by the DNeasy Tissue or Blood Kit (Qiagen), respectively, following the standard protocols, and then fragmented to a size ranging from 200 bp to 400 bp using Covaris S2 SonoLAB (Covaris). The QIAamp Circulating Nucleic Acid Kit (Qiagen) was used to extract ctDNA from the plasma. DNA concentrations were determined by the Qubit dsDNA HS Assay Kit (Life Technologies).

eMethods 5. Library Preparation, Target Capture and DNA Sequencing

gDNA libraries were produced by the KAPA Hyper Prep Kit (KAPA Biosystems) according to the manual. The ctDNA libraries were prepared by the Accel-NGS 2S Plus DNA Library Kit (SWIFT) with unique identifiers (UIDs, also called barcodes) to tag individual DNA molecules. The concentrations and size distributions of the libraries were respectively analyzed by Qubit and Caliper.

One to four libraries with different sample indexes were first pooled together to a total DNA amount of 1 µg. The pooled DNAs were mixed with 2 µl of DNA blocker (Integrated DNA Technologies) and 5 µl

© 2018 Wang Z et al. *JAMA Oncol.*

of human Cot-1 DNA (Invitrogen), and then dried using a vacuum concentrator (ThermoFisher). The dried mixture was dissolved in 15 μl hybridization buffer supplied by the hybridization of xGen Lockdown Probes (Integrated DNA Technologies), and thereafter, the IDT xGen Human Exome Research Panel kit and a customized set of biotinylated DNA probes were separately used to capture targeted DNAs for FFPE gDNA and plasma ctDNA following the standard protocols. The captured DNAs were then amplified by PCR, and the final DNA concentrations and sizes were respectively measured by Qubit and Caliper.

The captured libraries for FFPE gDNA were loaded into the HiSeq X (Illumina) for 150 bp paired-end sequencing, and the captured libraries for plasma ctDNA were loaded into the NextSeq 500 (Illumina) for 75 bp paired-end sequencing according to the manufacturer's instructions.

eMethods 6. WES Analysis Pipeline

Raw data (fastq file) with paired samples (FFPE and its normal control) were aligned to the human genome (hg19) using BWA Aligner v0.7.12. PCR duplicate reads were removed, and sequence metric collection was performed using Picard v1.130 (https://github.com/broadinstitute/picard/releases/tag/1.130) and SAMtools v1.1.19. Variant calling was performed in the targeted region. Somatic single nucleotide variants (SNVs) were called using muTect v1.1.7 (https://github.com/broadinstitute/mutect) and somatic indels were called using Pindel v0.2.5a8 (http://gmt.genome.wustl.edu/packages/pindel). All raw variants were then filtered by an automated false positive filtering pipeline to guarantee sensitivity and specificity above an allele frequency of 5%. SNPs were filtered by dbSNP, 1000g and ESP6500 (population frequency >0.015). TMB was defined as the sum of missense, stop loss, in-frame and frameshift mutations in protein coding regions.

eMethods 7. bTMB Detection Pipeline

Our bTMB detection is based on the ctDNA variant-calling method, with integrated digital barcodes to tag the individual DNA molecules. Such barcodes enable the precise molecular tracking, making it possible to distinguish authentic somatic mutations arising in vivo from artifacts introduced ex vivo.[3] The variant-calling pipeline was developed according to mapping information from BWA Aligner. To improve specificity, especially for variants with low allele frequency in the ctDNA, we developed a filtering model based on the binomial test and determined optimal thresholds for the different parts of error generation using our own training data.[4] The filtering model contains error filters at different phases of noise generation, including sample conservation, wet experiment and data analysis. Therefore, in the filtering model, background error correction, strand bias, base quality, mapping quality, short tandem repeat regions and low-quality mapping ratio are considered.

bTMB was defined as the number of somatic SNVS and indels in examined coding region. All SNVs and indels in the coding region of targeted genes, including missense, silent, stop gain, stop loss, in-frame and frameshift mutations, are initially considered. Known germline SNVs, defined as population frequency more than 0.015, in dbSNP, 1000 genome, and ESP6500 were filtered. Variants with allele frequencies more than 30%, which are more likely germline mutations, were not counted.

© 2018 Wang Z et al. *JAMA Oncol.*

eFigure 1. Flow Diagram of the Study. RGP: Randomized Gene Panel.

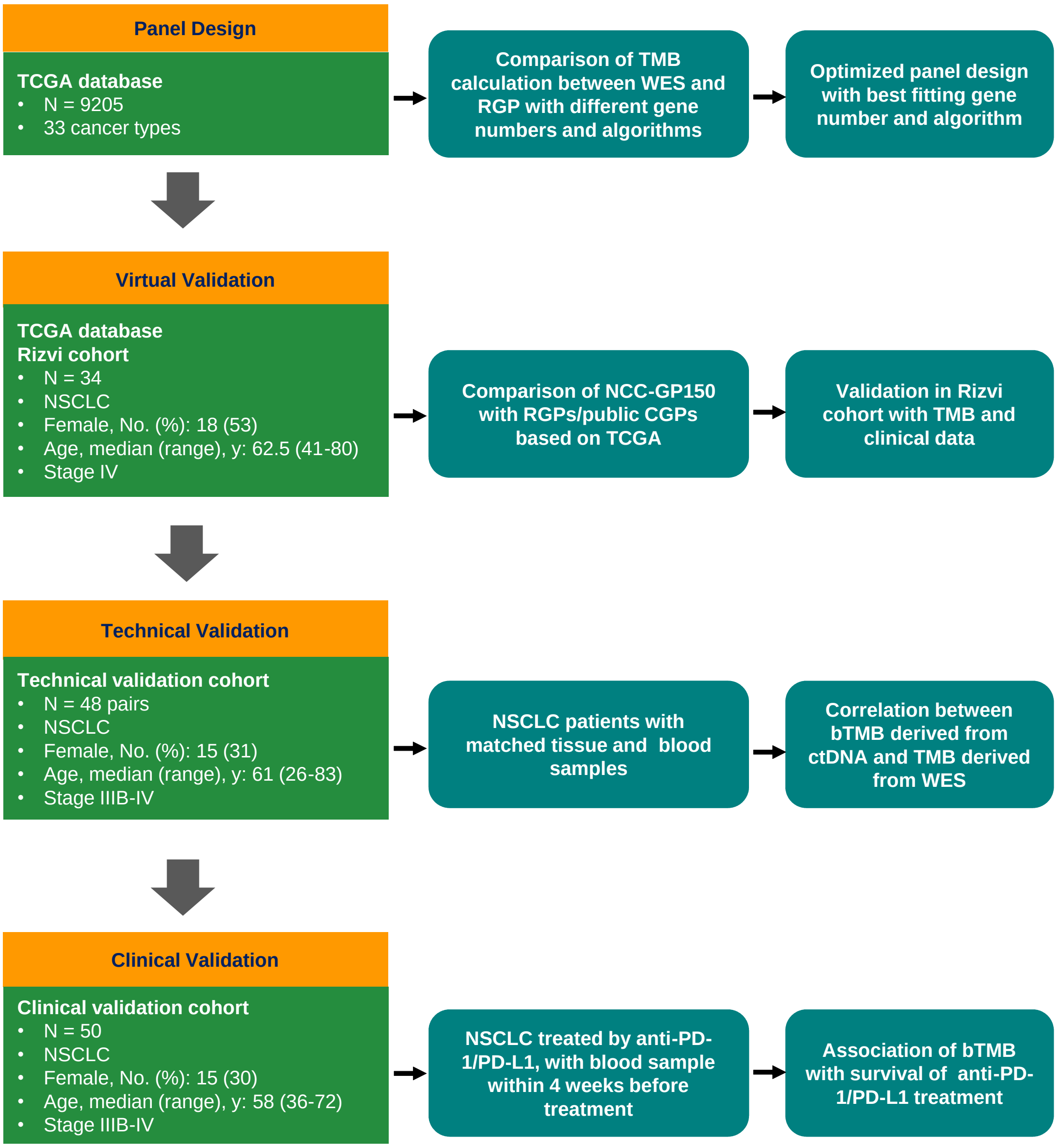

© 2018 Wang Z et al. *JAMA Oncol.*

eFigure 2. Flow Diagram for the Technical and Clinical Validation Cohorts.

(A) Patient disposition of 48 patients used for technical validation. (B) Patient disposition of 50 patients used for clinical validation.

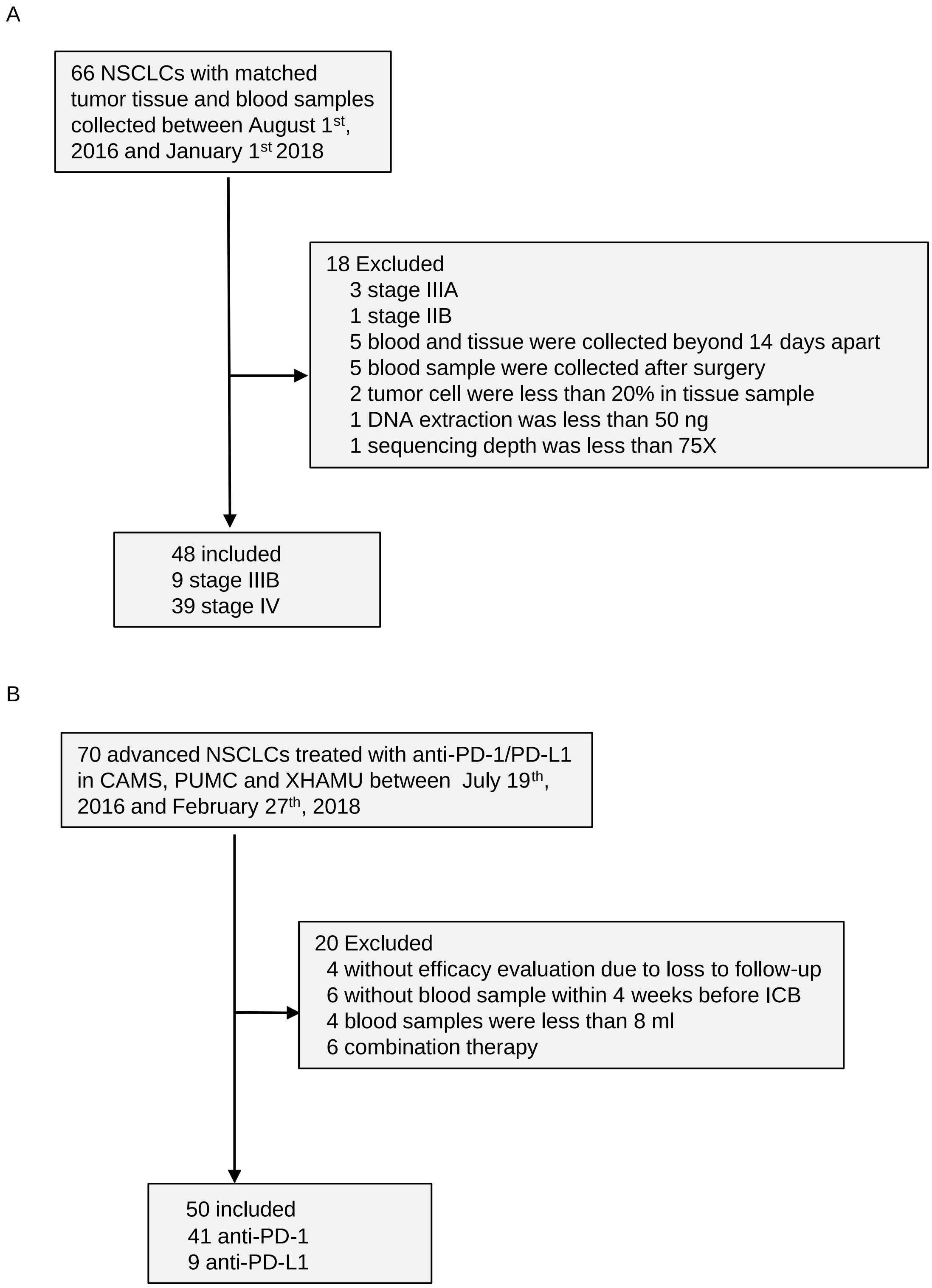

© 2018 Wang Z et al. *JAMA Oncol.*

eFigure 3. Venn Diagram and Flow Diagram Showing the Numbers and Percentages of Overlapping Gene Numbers Among NCC-GP150, F1CDx, MSK-IMPACT, and Guardant360. The overlapped gene numbers and percentages of NCC-GP150 with F1CDx, MSK-IMPACT, and Guardant360 are 124 (82.7%), 130 (86.7%), and 67 (44.7%), respectively. Those for Guardant360 with F1CDx and MSK-IMPACT are 69 (94.5%) and 73 (100%), respectively, and 259 (79.9%) between F1CDx and MSK-IMPACT.

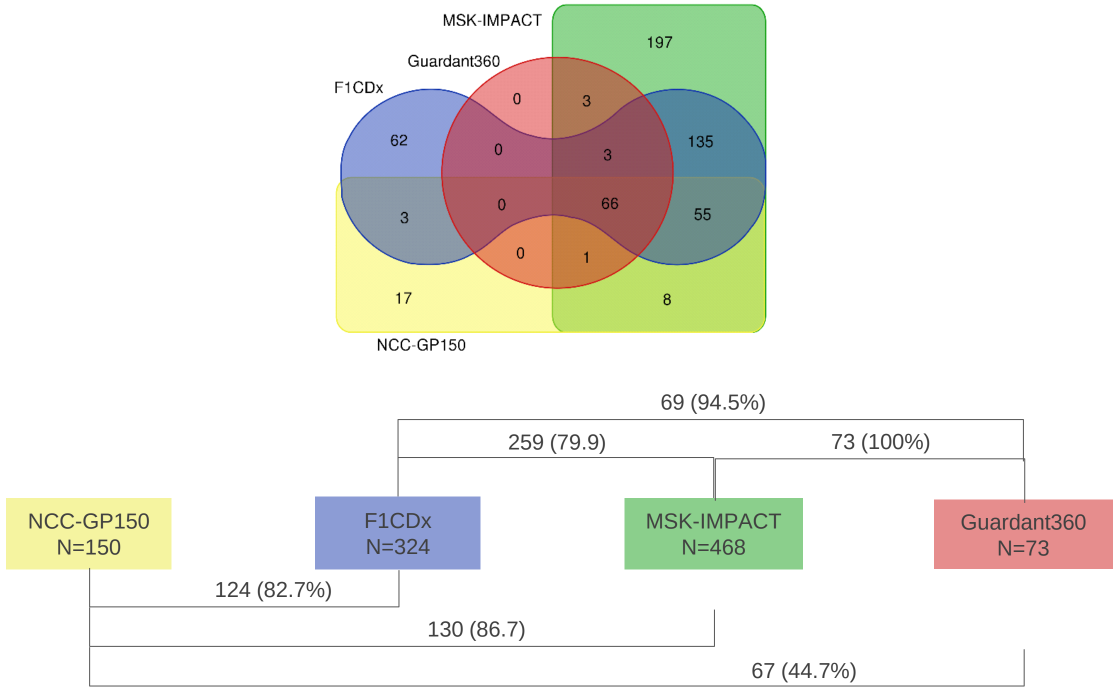

© 2018 Wang Z et al. *JAMA Oncol.*

eFigure 4. Comparison of Panel Performance Between TCGA NSCLC Populations With and Without EGFR or KRAS Driver Mutation.

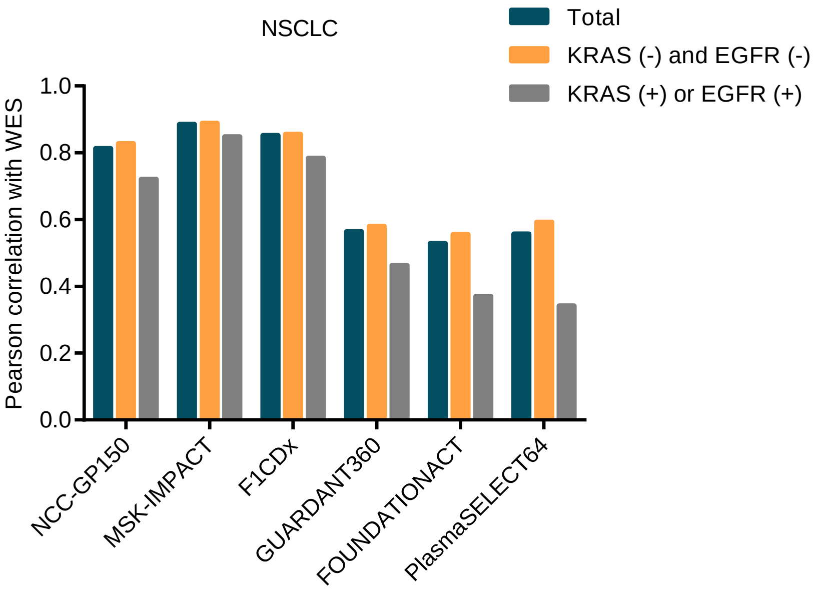

© 2018 Wang Z et al. *JAMA Oncol.*

eFigure 5. Technical validation of the association between bTMB estimated by NCC-GP150 and WES. (A) Matched tissue/blood TMB comparison. (B) Agreement between bTMB and TMB statuses.

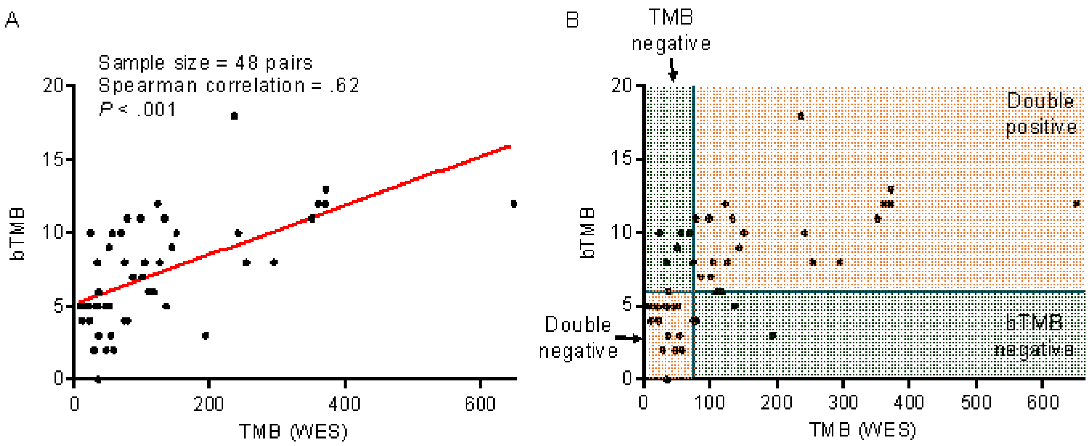

© 2018 Wang Z et al. *JAMA Oncol.*

eFigure 6. Comparison of Progression-Free Survival Between Patients in the bTMB-H and bTMB-L Groups With Different Cut-Points for bTMB.

Cut-points	Anti-PD-1/PD-L1 therapy: bTMB ≥ cut-points N (%)	PFS HR (95% CI)	*P*
4	40 (80%)	0.73 (0.31-1.72)	.47
5	36 (72%)	0.58 (0.27-1.26)	.17
6	28 (56%)	0.39 (0.18-0.84)	.02
7	25 (50%)	0.46 (0.21-1.01)	.05
8	21 (42%)	0.43 (0.19-0.98)	.05
9	19 (38%)	0.59 (0.26-1.35)	.21
10	14 (28%)	0.70 (0.28-1.72)	.43

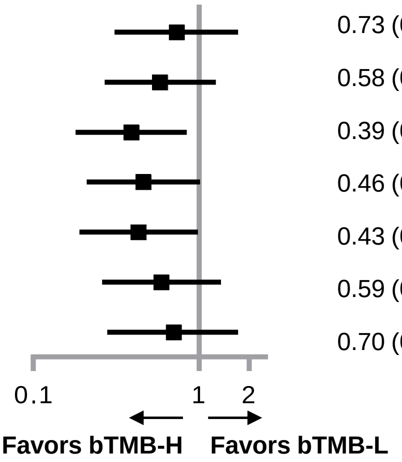

© 2018 Wang Z et al. *JAMA Oncol.*

eFigure 7. Comparison of Progression-Free Survival Between bTMB-H and bTMB-L With Different Cut-Points for bTMB in Patients With Anti-PD1/PD-L1 Therapy as First- or Second-Line Treatment.

Cut-points	Anti-PD-1/PD-L1 therapy		
	bTMB≥ cut-points N (%)	PFS HR (95% CI)	*P*
4	25 (76%)	0.38 (0.12-1.18)	.10
5	22 (67%)	0.25 (0.08-0.75)	.01
6	18 (55%)	0.08 (0.02-0.36)	.001
7	16 (48%)	0.12 (0.03-0.55)	.006
8	15 (45%)	0.15 (0.03-0.69)	.02
9	13 (39%)	0.24 (0.05-1.07)	.06
10	9 (27%)	0.21 (0.03-1.61)	.13

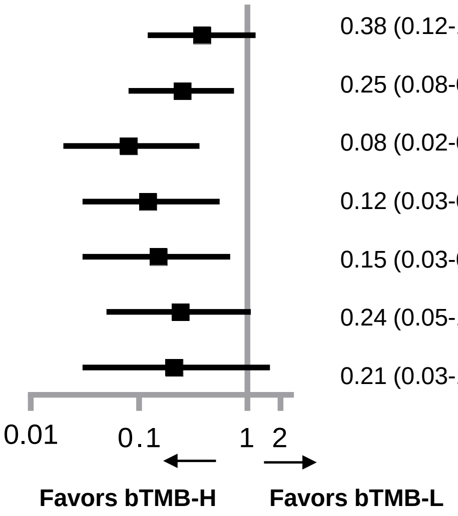

© 2018 Wang Z et al. *JAMA Oncol.*

eFigure 8. Association Between bTMB and Clinical Outcomes in Patients With Anti-PD1/PD-L1 Therapy as First- or Second-Line Treatment. (A) Progression-free survival by bTMB status for ICB administered as a first- or second-line treatment. (B) Comparison of objective response rate between the bTMB-H and bTMB-L groups for ICB administered as a first- or second-line treatment. (C) Comparison of bTMB between nonresponse and response groups for ICB administered as a first- or second-line treatment.

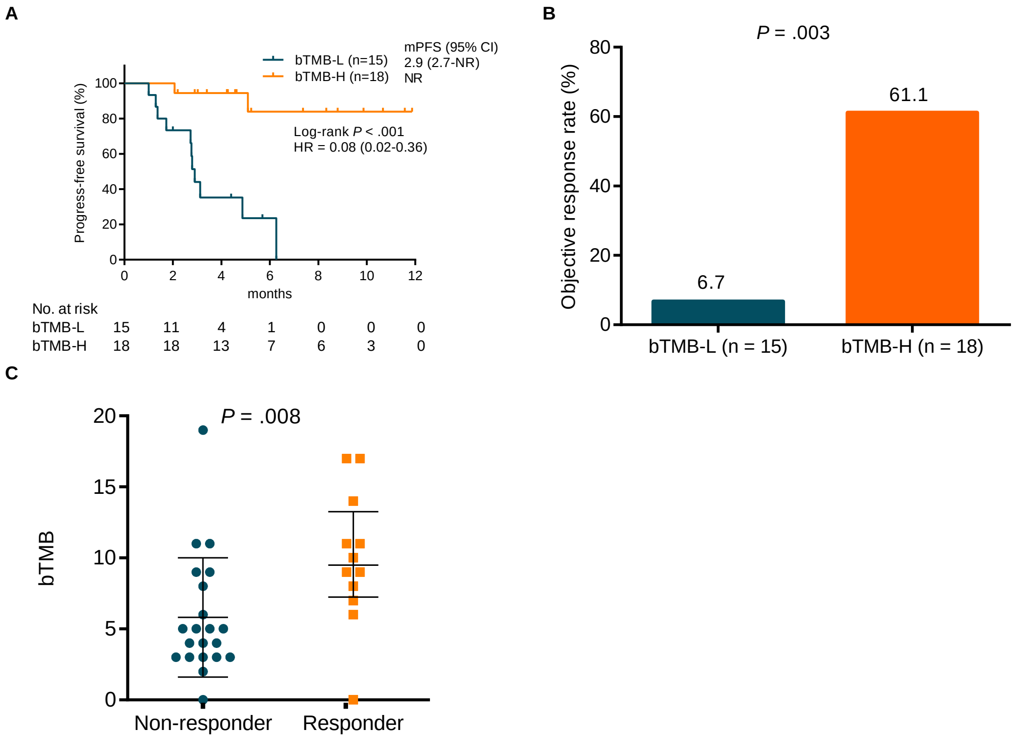

© 2018 Wang Z et al. *JAMA Oncol.*

eTable 1. Detailed List of Genes in the NCC-GP150 Panel.

Gene list
ACVR2A AKT1 AKT2 ALK APC AR ARAF ARID1A ARID2 ATM ATR AXIN1 BARD1 BCL2L11 BIRC5 BRAF BRCA1 BRCA2 BRIP1 C11orf30 CBL CCND1 CCND2 CCNE1 CD274 CDH1 CDK12 CDK4 CDK6 CDKN1B CDKN2A CHEK1 CHEK2 CREBBP CRKL CTNNB1 CYP2C19 CYP2D6 DDR2 DPYD EGFR EP300 EPHB1 ERBB2 ERBB3 ERBB4 ERRFI1 ESR1 EZH2 FAM135B FAT1 FBXW7 FGF19 FGFR1 FGFR2 FGFR3 FLT1 FLT3 FLT4 GATA3 GLI3 GNA11 GNAQ GNAS HNF1A HRAS IDH1 IDH2 IRS2 JAK2 JAK3 KDR KEAP1 KIT KMT2A KRAS LRP1B MAP2K1 MAP2K2 MAP2K4 MAP3K1 MCL1 MET MLH1 MRE11A MSH2 MSH6 MTOR MYC MYCL MYCN NF1 NFE2L2 NKX2-1 NOTCH1 NOTCH2 NOTCH3 NRAS NRG1 NRG3 NTRK1 NTRK2 NTRK3 PALB2 PDCD1LG2 PDGFRA PDGFRB PIK3CA PIK3R1 PREX2 PTCH1 PTEN PTK2 PTPN11 RAD50 RAF1 RB1 RBM10 RET RICTOR RIT1 RNF43 ROS1 RUNX1T1 SETD2 SLIT2 SMAD2 SMAD3 SMAD4 SMARCA2 SMARCA4 SMO SOX2 SPEN SPTA1 SRC STK11 TBX3 TCF7L2 TERT TGFBR2 TP53 TPMT TSC1 TSC2 UGT1A1 VEGFA VHL ZNF217 ZNF703

© 2018 Wang Z et al. *JAMA Oncol.*

eTable 2. Summary of Pearson Correlation Between Different Public Panels and WES for Different Tumor Types.

Tumor Type	Abbreviation in TCGA	N	MSK IMPACT	F1CDx	NCC-GP150	Guardant 360	PlasmaSELECT64	FOUNDATIONACT
Pancreatic adenocarcinoma	paad	150	0.9991	0.9986	0.9971	0.9916	0.9917	0.9890
Skin Cutaneous Melanoma	skcm	368	0.9943	0.9921	0.9900	0.9754	0.9767	0.9674
Prostate adenocarcinoma	prad	499	0.9860	0.9826	0.9621	0.8928	0.8852	0.8772
Uterine Corpus Endometrial Carcinoma	ucec	248	0.9844	0.9843	0.9865	0.9619	0.9594	0.9553
Stomach adenocarcinoma	stad	395	0.9839	0.9725	0.9578	0.8884	0.9078	0.8717
Colon adenocarcinoma and Rectum adenocarcinoma	coadread	223	0.9813	0.9696	0.9842	0.9373	0.9444	0.9254
Uterine Carcinosarcoma	ucs	57	0.9735	0.9650	0.9618	0.8799	0.8799	0.8192
Breast invasive carcinoma	brca	982	0.9370	0.9163	0.8608	0.6491	0.6013	0.5295
Lymphoid Neoplasm Diffuse Large B-cell Lymphoma	dlbc	48	0.9347	0.8710	0.8737	0.6739	0.7057	0.6889
Thymoma	thym	123	0.9264	0.9243	0.8643	0.5988	0.5089	0.5340
Lung adenocarcinoma	luad	230	0.9154	0.8400	0.8302	0.6578	0.6229	0.6087
Esophageal carcinoma	esca	185	0.9053	0.8615	0.7937	0.5114	0.5738	0.4161
Liver hepatocellular carcinoma	lihc	373	0.9023	0.8575	0.7625	0.4234	0.4645	0.4290
Adrenocortical carcinoma	acc	90	0.8912	0.9268	0.9444	0.8179	0.7971	0.7779
Lung squamous cell carcinoma	lusc	178	0.8903	0.8367	0.8253	0.5602	0.5074	0.5888
Cervical squamous cell carcinoma and endocervical adenocarcinoma	cesc	194	0.8885	0.8578	0.7995	0.6210	0.7147	0.6402
Non-Small Cell Lung Cancer	nsclc	1144	0.8851	0.8517	0.8133	0.5639	0.5568	0.5285
Head and Neck squamous cell carcinoma	hnsc	512	0.8825	0.8435	0.7577	0.5049	0.4991	0.4851
Kidney renal clear cell carcinoma	kirc	451	0.8738	0.8620	0.7604	0.5535	0.5922	0.6071
Sarcoma	sarc	247	0.8731	0.8117	0.7904	0.5501	0.4624	0.4430
Bladder Urothelial Carcinoma	blca	130	0.8717	0.8119	0.7320	0.5334	0.5109	0.5340

© 2018 Wang Z et al. *JAMA Oncol.*

Uveal Melanoma	uvm	80	0.8693	0.8225	0.7031	0.5246	0.6254	0.4343
**Tumor Type**	**Abbreviation in TCGA**	**N**	**MSK IMPACT**	**F1CDx**	**NCC-GP150**	**Guardant 360**	**PlasmaSELECT64**	**FOUNDATIONACT**
Kidney Chromophobe	kich	66	0.8685	0.8551	0.7435	0.5143	0.5802	0.3114
Cholangiocarcinoma	chol	35	0.8578	0.7781	0.6677	0.2313	0.5071	0.1585
Brain Lower Grade Glioma	lgg	286	0.4890	0.3094	0.2123	0.1307	0.1116	0.0360
Testicular Germ Cell Tumors	tgct	155	0.4447	0.3295	0.1761	0.0231	0.0031	0.0047
Kidney renal papillary cell carcinoma	kirp	282	0.3729	0.2497	0.1696	0.0821	0.0698	0.0462
Glioblastoma multiforme	gbm	290	0.3237	0.1772	0.1777	0.1047	0.1081	0.1076
Ovarian serous cystadenocarcinoma	ov	316	0.3200	0.1955	0.1631	0.0792	0.0658	0.0495
Thyroid carcinoma	thca	401	0.2791	0.1841	0.0790	0.0244	0.0407	0.0350
Acute Myeloid Leukemia	laml	197	0.2039	0.1557	0.1097	0.0677	0.0327	0.0550
Pheochromocytoma and Paraganglioma	pcpg	184	0.1253	0.0706	0.0549	0.0026	0.0089	0.0029
Mesothelioma	meso	82	0.0922	0.0811	0.0164	0.0164	0.0164	0.0164
All	all	9201	0.9731	0.9640	0.9607	0.8792	0.8953	0.8645

© 2018 Wang Z et al. *JAMA Oncol.*

eTable 3. Detailed Clinicopathologic Features of 48 NSCLC Patients for bTMB Technical Validation.

**Characteristics**	**All patients (N = 48)**
Age at initiation of ICB, median (IQR), y	61 (53-68)
Sex, N (%)	
Male	33 (68.8)
Female	15 (31.2)
Histologic type, N (%)	
Squamous cell carcinoma	12 (25.0)
Non-squamous cell carcinoma	33 (68.8)
Missing	3 (6.2)
Stage, N (%)	
IIIB	9 (18.8)
IV	39 (81.2)
bTMB, median (IQR)	7 (5-10)
TMB (WES), median (IQR)	75 (34-141)

© 2018 Wang Z et al. *JAMA Oncol.*

eTable 4. Summary of Sensitivity, Specificity and Youden's Index at Different Cutoffs for bTMB-H in the Matched Tissue/Blood TMB Comparison.

bTMB	sensitivity	specificity	Youden's index
≥ 4	0.96	0.25	0.21
≥ 5	0.92	0.38	0.30
≥ 6	0.88	0.71	0.59
≥ 7	0.80	0.75	0.55
≥ 8	0.71	0.75	0.46
≥ 9	0.54	0.83	0.37
≥ 10	0.50	0.88	0.38
≥ 11	0.42	1.00	0.42

© 2018 Wang Z et al. *JAMA Oncol.*

eTable 5. Detailed Clinicopathologic Features of 50 NSCLC Patients for bTMB Clinical Validation by bTMB Status.

Characteristics	bTMB < 6 (N = 22)	bTMB ≥ 6 (N = 28)	*P*
Age at initiation of ICB, median (IQR), y	56 (7)	59 (8)	.11
Sex, N (%)			.07
Male	12 (54.5)	23 (82.1)	
Female	10 (45.5)	5 (17.9)	
Histologic type, N (%)			.61
Squamous cell carcinoma	7 (31.8)	12 (42.9)	
Non-squamous cell carcinoma	15 (68.2)	16 (57.1)	
No. of Metastatic sites, N (%)			.04
0	1 (4.5)	0	
1	10 (45.5)	6 (21.4)	
≥ 2	11 (50.0)	22 (78.6)	
ECOG performance status, N (%)			.44
0-1	20 (90.9)	23 (82.1)	
2-4	2 (9.1)	5 (17.9)	
Smoking status, N (%)			.39
Current	8 (36.4)	14 (50.0)	
Former	1 (4.5)	3 (10.7)	
Never	13 (59.1)	11 (39.3)	
LDH, median (IQR)			.56
< 250 U/L	16 (72.7)	17 (60.7)	
≥ 250 U/L	6 (27.3)	11 (39.3)	
PD-L1 status, median (IQR)			.97
< 1%	6 (27.3)	8 (28.6)	
≥ 1%	11 (50.0)	13 (46.4)	
Missing	5 (22.7)	7 (25.0)	
Immune checkpoint blockade therapy, N (%)			.44
Anti-PD-1	20 (90.9)	22 (78.6)	
Anti-PD-L1	2 (9.1)	6 (21.4)	
Treatment lines, N (%)			1.00
1-2	15 (68.2)	18 (64.3)	
≥ 3	7 (31.8)	10 (35.7)	
Hospital sites, N (%)			1.00
Cancer Hospital CAMS	15 (68.2)	18 (64.3)	
Pecking University Cancer Hospital	5 (22.7)	7 (25.0)	
Xinqiao Hospital Army Medical University	2 (9.1)	3 (10.7)	

P values for continuous variables were calculated with Student's t test and categorical variables with chi-square test or Fisher exact test.

SI conversion factors: To convert lactate dehydrogenase to microkatals per liter, multiply by 0.0167.

© 2018 Wang Z et al. *JAMA Oncol.*

eReferences

1. Wang Z, Zhao J, Wang G, et al. Comutations in DNA Damage Response Pathways Serve as Potential Biomarkers for Immune Checkpoint Blockade. *Cancer Res.* 2018;78(22):6486-6496. doi: 10.1158/0008-5472.CAN-18-1814.
2. Eisenhauer EA, Therasse P, Bogaerts J, et al. New response evaluation criteria in solid tumours: revised RECIST guideline (version 1.1). *Eur J Cancer.* 2009;45(2):228-247. doi: 10.1016/j.ejca.2008.10.026.
3. Newman AM, Lovejoy AF, Klass DM, et al. Integrated digital error suppression for improved detection of circulating tumor DNA. *Nat Biotechnol.* 2016;34(5):547-555. doi: 10.1038/nbt.3520.
4. Yang N, Li Y, Liu Z, et al. The characteristics of ctDNA reveal the high complexity in matching the corresponding tumor tissues. *BMC Cancer.* 2018;18(1):319. doi: 10.1186/s12885-018-4199-7.

© 2018 Wang Z et al. *JAMA Oncol.*
